# Supplementary material for: Analysis of the Level of Plasmid-Derived mRNA in the Presence of Residual Plasmid DNA by Two-Step Quantitative RT-PCR
Source: Methods Protoc. 2020 May 23;3(2):40. doi: 10.3390/mps3020040 (PMC7359704; doi:10.3390/mps3020040)
Supplement: Supplementary file 1 [file mps-03-00040-s001.pdf]

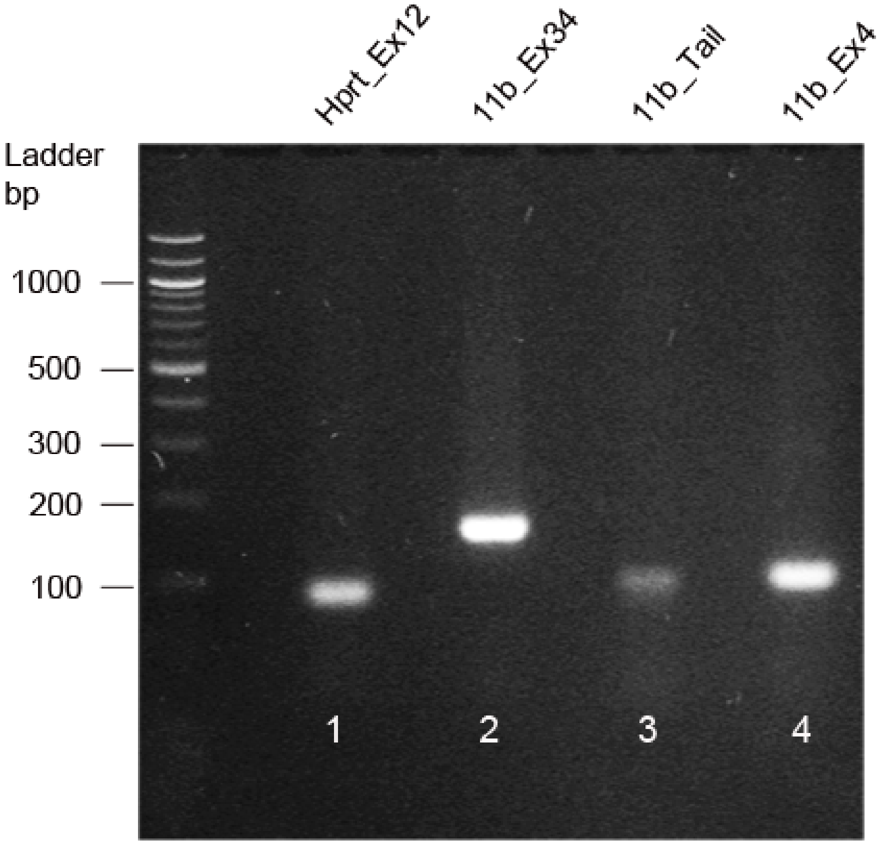

Supplementary Figure 1: Agarose gel electrophoresis (3%) of the PCR products from the cDNA of *Pex11β* vector-transfected cells (6 h post-transfection). The total RNA was reversely transcribed using random hexamers (random RT) or the nonsense-tail RT primer (nonsense-tail RT). Lane 1: random RT, the Hprt_Ex12 PCR primer product of 88 bp; Lane 2: random RT, the 11b_Ex34 PCR primer product of 158 bp; Lane 3: nonsense-tail RT, the 11b_Tail PCR primer product of 80 bp; Lane 4: random RT, the 11b_Ex4 PCR primer product of 96 bp. The 100-bp DNA molecular weight marker is shown on the left.
